# Pulsed Stimuli Elicit More Robust Multisensory Enhancement than Expected

**DOI:** 10.3389/fnint.2017.00040

**Published:** 2018-01-04

**Authors:** Eva C. Bach, John W. Vaughan, Barry E. Stein, Benjamin A. Rowland

**Affiliations:** Department Neurobiology and Anatomy, Wake Forest School of Medicine, Winston-Salem, NC, United States

**Keywords:** multisensory integration, sensory neuroscience, multisensory enhancement, dynamic stimuli, superior colliculus (SC)

## Abstract

Neurons in the superior colliculus (SC) integrate cross-modal inputs to generate responses that are more robust than to either input alone, and are frequently greater than their sum (superadditive enhancement). Previously, the principles of a real-time multisensory transform were identified and used to accurately predict a neuron's responses to combinations of brief flashes and noise bursts. However, environmental stimuli frequently have more complex temporal structures that elicit very different response dynamics than previously examined. The present study tested whether such stimuli (i.e., pulsed) would be treated similarly by the multisensory transform. Pulsing visual and auditory stimuli elicited responses composed of higher discharge rates that had multiple peaks temporally aligned to the stimulus pulses. Combinations pulsed cues elicited multiple peaks of superadditive enhancement within the response window. Measured over the entire response, this resulted in larger enhancements than expected given enhancements elicited by non-pulsed (“sustained”) stimuli. However, as with sustained stimuli, the dynamics of multisensory responses to pulsed stimuli were highly related to the temporal dynamics of the unisensory inputs. This suggests that the specific characteristics of the multisensory transform are not determined by the external features of the cross-modal stimulus configuration; rather the temporal structure and alignment of the unisensory inputs is the dominant driving factor in the magnitudes of the multisensory product.

## Introduction

Brains integrate information across their multiple sensory systems to enhance the detection, localization, and identification of external stimuli, consequently improving perceptual and behavioral responses to them (see reviews in Stein, 2012). One circuit in which this occurs is the cat midbrain superior colliculus (SC), a topographically-organized sensorimotor structure that detects and then facilitates orientation toward environmental events (Sprague and Meikle, 1965; Stein et al., 1976, 1989; Stein and Clamann, 1981; Munoz et al., 1991; Paré et al., 1994; Lomber et al., 2001; Burnett et al., 2004; Guillaume and Pélisson, 2006; Rowland et al., 2007a; Gingras et al., 2009). Within the SC, afferents relaying independent signals from multiple sensory regions (visual, auditory, and somatosensory) converge onto target multisensory neurons that transform their inputs to integrated multisensory responses. When these signals relay spatiotemporally concordant cross-modal cues, SC multisensory responses are greater in magnitude than those elicited by either cue individually (Meredith and Stein, 1983; Wallace et al., 1998; Jiang et al., 2001; Perrault et al., 2005; Stanford et al., 2005; Alvarado et al., 2007a; Rowland and Stein, 2008). The neural products of multisensory integration, measured by changes in the impulse counts or firing rates of individual neurons, have been documented in a wide variety of contexts, conditions, circuits, and species (Meredith and Stein, 1983; King and Palmer, 1985; Meredith et al., 1987; Binns and Salt, 1996; Wallace and Stein, 1996; Bell et al., 2001; Wallace et al., 2004; Ghazanfar and Schroeder, 2006; Jain and Shore, 2006; Nagy et al., 2006; Avillac et al., 2007; Bizley et al., 2007; Lakatos et al., 2007; Romanski, 2007; Winkowski and Knudsen, 2007; Bizley and King, 2008; Reches and Gutfreund, 2009; Zahar et al., 2009; Fetsch et al., 2012; Lippert et al., 2013; Reig and Silberberg, 2014; Ishikawa et al., 2015; Costa et al., 2016; Felch et al., 2016; Kardamakis et al., 2016; Bieler et al., 2017; Truszkowski et al., 2017). Recent efforts, directed at understanding the neural *process* that generates these products in the SC (i.e., the multisensory transform), have suggested two principles for its operation (Miller et al., 2015, 2017; see also Felch et al., 2016). The first is that cross-modal inputs are integrated as soon as they arrive at the target neuron (see also Rowland et al., 2007b). The second is that responses are influenced by a delayed, calibrating inhibitory dynamic that is stronger than that affecting unisensory responses (Miller et al., 2015). Together, these principles explain why the products of multisensory integration can be exquisitely sensitive to the temporal dynamics and alignments of the unisensory inputs. As demonstrated by Miller et al. (2017), they can be used to construct a neurocomputational model (continuous-time multisensory model, CTMM) to accurately predict the millisecond-by-millisecond responses of individual neurons to a cross-modal stimulus complex given only knowledge of how the neuron responds to each of the individual stimulus components and how they are arranged in time.

One practical consequence of understanding the multisensory transform is that it provides a framework within which to anticipate the products of integration in different conditions. However, at present our understanding of this transform has only been evaluated *post*-*hoc* in a very limited experimental context: the responses of SC neurons to sustained stimuli. Since the principles of this transform place such great importance on the temporal dynamics of the unisensory inputs, we sought to evaluate whether these principles would hold for SC responses to cues with a more complex temporal structure. This was the goal of the present effort.

## Methods

### Animals

Protocols for all animal procedures were in compliance with standards set forth by the National Institutes of Health's “Guide for the Care and Use of Laboratory Animals, Ed 8.” All procedures were approved by the Animal Care and Use Committee of Wake Forest School of Medicine and the Association for the Assessment and Accreditation of Laboratory Care International.

### Surgical procedures

Each cat (*n* = 4, 2 males and 2 female) was transported to the surgical preparation room and injected with ketamine hydrochloride (25–30 mg/kg, IM) and acepromazine maleate (0.1 mg/kg, IM). The scalp was shaved and cleaned and the animal was intubated. It was then transferred to the surgical suite where it was placed in a stereotaxic head holder, artificially respired, and anesthesia was induced and maintained throughout with isoflurane (1.5–3.0%). Vital signs (expired CO2, blood pressure and heart rate) were monitored (VetSpecs VSM7) throughout, and body temperature was maintained with a heating pad. An incision was made in the scalp, the skin and muscle were retracted, a craniotomy was made to provide access to the SC, and a stainless steel recording chamber was affixed to the skull over the opening (McHaffie and Stein, 1983). Upon completing the recording well-implantation the anesthetic was discontinued, the animal was extubated, and prophylactic antibiotics (5 mg/kg enrofloxacin, IM) and analgesics (0.01 mg/kg buprenorphine, IM) were administered. Once the animal regained mobility it was returned to its home pen. Analgesics (0.01 mg/kg buprenorphine, IM) were given twice daily for up to 3 days post-surgery as needed. Animals were allowed to recover from surgery for 7 days prior to commencement of weekly recording sessions.

### Recording procedures

For each session the animal was anesthetized with ketamine hydrochloride (25–30 mg/kg, IM) and acepromazine maleate (0.1 mg/kg, IM), intubated, and artificially respired. A catheter was placed into the medial femoral vein. To preclude movement artifacts, prevent ocular drift, and maintain the pinnae in place, neuromuscular blockade was induced with an initial dose of rocuronium (0.7 mg/kg, IV). Anesthesia, paralysis, and hydration were maintained with ketamine hydrochloride (5–10 mg/kg/h), rocuronium (1–3 mg/kg/h), and 5% dextrose in sterile saline delivered IV via an infusion pump (rate = 2–4 ml/h). Vital signs were monitored throughout the duration of the recording (VetSpecs VSM6). To focus the eyes on the screen and prevent corneal drying, contact lenses were placed in the eyes, and the eye ipsilateral to the SC being studied was occluded.

Parylene coated tungsten electrodes (impedance: 1–4 MΩ) were lowered into the intermediate and deep (i.e., multisensory) layers of the SC. Single neuron impulses were amplified using a FHC X-cell amplifier with band pass filters set between 300 and 5,000 Hz. Neuronal impulses were electronically discriminated and the raw traces were digitized at 20 KHz. Isolated single neurons individually responsive to both visual and auditory stimuli were targeted (*overt* multisensory neurons, see Yu et al., 2010; Xu et al., 2012). Visual stimuli consisted of flashed (fV) or moving bars of light back-projected on a screen 45 cm in from of the animal. Contrast of these stimuli was adjusted to produce low unisensory response efficacies conducive for observing multisensory enhancement. Auditory stimuli (A) were broadband (0.1–12 kHz) noise bursts with a square wave envelope projected from speakers positioned on a mobile hoop 15° apart and 15 cm from the animal's head. Visual and auditory receptive fields were mapped for each neuron using standard techniques (e.g., see Alvarado et al., 2007b; Yu et al., 2010) and stimuli were presented within the best areas.

Stimuli (light patches, light bars, and noise bursts) were presented with two different temporal structures, one constant (“sustained”) and one pulsed. The “sustained” visual patches (fVs), moving visual bars (mVs), and auditory bursts (As) were 250 ms in total duration. The “pulsed” stimuli consistent of three iterations (“pulses”) of the visual patches (fVp), illuminations of the moving visual bar (mVp), or noise bursts (Ap), each 50 ms in duration, with a 50 ms gap between each (250 ms total duration). Neurons were tested with the stimuli individually and in combinations in a factorial design. Thus, there were 6 (fVs, mVs, As, fVp, mVp, Ap) modality-specific tests and 8 cross-modal tests for each neuron [(fVs, mVs, fVp, mVp) × (As, Ap)] that were presented in pseudo-random order (20 trials/condition) with an inter-stimulus interval of 6 sec. For cross-modal sensory conditions, we limited our analysis to a stimulus onset asynchrony (SOA) in which the visual stimulus always preceded the auditory stimulus by 25 ms (cf. Miller et al., 2015).

Upon completion of the recording session, the anesthetic and neuromuscular block were terminated. When appropriate, based on the return of breathing control and mobility, the animal was extubated and placed in a carrier cage for close monitoring. Once the animal regained sternal recumbency and locomotion, it was returned to its home pen.

### Data analysis

Impulse rasters for the response to each cue were recorded and processed with a 1 ms temporal resolution. A 3-step geometric method was used to identify the beginning and end of the total response window (Rowland et al., 2007c). Impulse rasters were additionally blocked into three windows: an early window (30–100 ms after stimulus onset), a middle window (100–200 ms after stimulus onset), and a late window (200–350 ms after stimulus onset). The first window was chosen to include as little spontaneous activity as possible (beginning just before the earliest possible response onset, stimulus onset + 30 ms) but no activity that would be associated with the second stimulus (ending at second stimulus onset). To prevent gaps, the second window spanned between the second stimulus onset and the third stimulus onset. The third window began at the third stimulus onset and attempted to capture the tail of the response. The dependent variable of principal interest in each window was the mean stimulus-driven firing rate (measured in impulses/s, “Hz”), calculated by averaging the number of impulses within the window across trials, dividing by the window size, and subtracting the spontaneous firing rate observed prior to stimulus onset. In addition, the instantaneous firing rate trace for each response was calculated by convolving the raster with a narrow (8 ms std) Gaussian kernel, averaging across trials, and subtracting the mean spontaneous firing rate observed in the 500 ms pre-stimulus window on each trial.

The responses of a neuron to each cross-modal stimulus complex (VA) were linked to its responses to the constituent visual (V) and auditory (A) stimuli. Multisensory enhancements were evaluated by comparing the multisensory responses to the linked unisensory responses; thus, each of these triads represented a “sample” for analysis. Comparisons were made between firing rates over the entire response window and within restricted windows of time. There were two principal metrics in these comparisons: multisensory enhancement (ME, proportionate difference between the multisensory and largest unisensory responses) and the additivity index (AI, proportionate difference between the multisensory and summed unisensory responses):

$$\begin{array}{lll} ME & = & 100\times \frac{VA-\text{max}\left(V,A\right)}{\text{max}\left(V,A\right)}\\ AI & = & 100\times \frac{VA-\left(V+A\right)}{\left(V+A\right)} \end{array}$$

For each evaluation, a multisensory response was deemed to be enhanced if the multisensory response magnitude was significantly elevated over the largest unisensory response magnitude in the sample (Meredith and Stein, 1986), evaluated across paired trials with Wilcoxon signed rank test. It was classified as superadditive if the multisensory response was significantly greater than the summed unisensory response magnitudes (z-test). The distribution of the expected summed unisensory response magnitudes for this evaluation was bootstrapped as previously described (Stanford et al., 2005).

Samples of responses were pooled across neurons based on the temporal structure of the stimuli in the cross-modal condition, of which there were four possible combinations (VsAs, VsAp, VpAs, and VpAp) after collapsing across visual stimulus type. Average response magnitudes, ME, and AI were calculated within and compared across these conditions using parametric tests. These groups were further pooled to isolate specific changes in the multisensory products associated with pulsed vs. sustained stimuli of each modality. For example, the effect of pulsed vs. sustained visual stimuli was evaluated by constructing a pool of all 4 cross-modal conditions in which the visual was pulsed (irrespective of the auditory structure) and comparing it with the pool of 4 conditions in which the visual was sustained. In addition, we highlighted for special recognition the conditions in which the V and A stimuli were either both sustained (the “sustained group,” VsAs) or both pulsed (the “pulsed group,” VpAp).

In addition, a correlational approach was used to confirm the relationship between the temporal dynamics of the multisensory and unisensory responses in the pooled groups as established previously for sustained stimuli (Miller et al., 2017). This analysis is a correlation calculated within a group (i.e., across neurons/samples) at each millisecond in time between the multisensory instantaneous firing rate traces and the V, A, and summed V+A instantaneous firing rate traces (aligned by stimulus onsets). The result is a R^2^ value at each moment in time indicating the proportion of variance in the multisensory response (across neurons in the group) explained by the unisensory response variance. This was evaluated here for groups of sustained and pulsed stimuli.

## Results

A total of 31 neurons were recorded in the multisensory (i.e., intermediate and deep) layers of the SC. Of this population 19 (61%) evidenced significant responses for both the visual and auditory modalities (*p* < 0.05) and significant multisensory enhancement (*p* < 0.05). This population of *overt enhancing* neurons was used in all subsequent analyses. Some neurons were tested multiple times with alternating background to stimulus contrast (light stimulus presented on dark background or alternated to present dark stimulus on light background),and all with two configurations of the visual cues, resulting in a total of 45 samples for each of the groups of cross-modal pairs (i.e., VsAs, VsAp, VpAs, VpAp).

**Figure 1:**
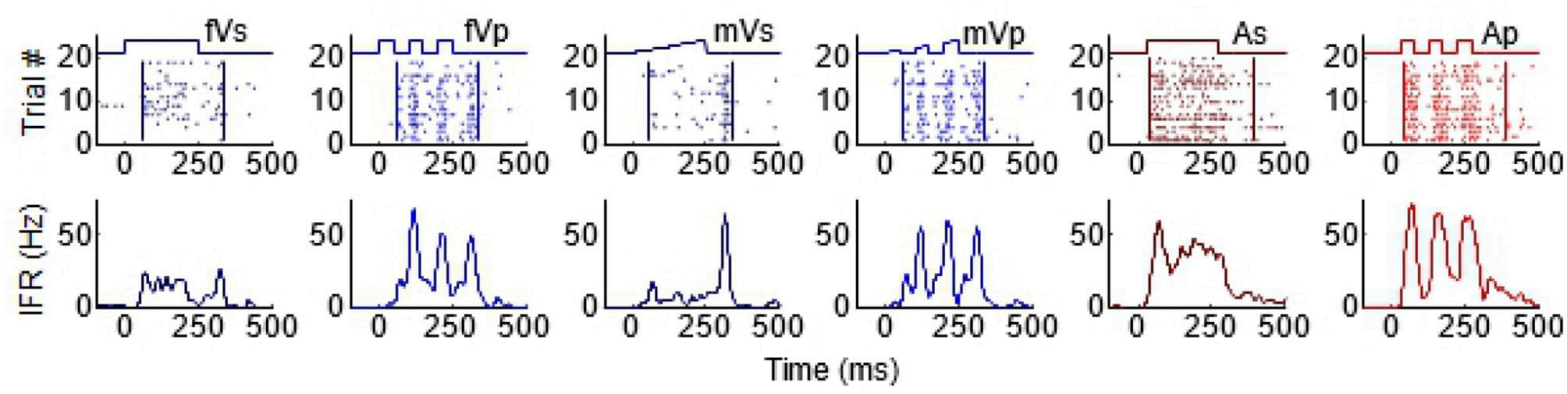
Temporal profiles of SC visual and auditory responses produced by sustained and pulsed stimuli. Each column illustrates the response to a different stimulus, from left to right: fVs, flashed visual sustained; fVp, flashed visual pulsed; mVs, moving visual sustained; mVp, moving visual pulsed; As, auditory sustained; Ap, auditory pulsed. The top row of plots illustrates raster displays for each response (each dot = 1 impulse). Traces above the raster display show the timing of each stimulus. The different stimulus dynamics evoked responses with very different temporal profiles and are summarized by the corresponding instantaneous firing rate (IFR) traces on the bottom row. Note that pulsed stimuli elicit responses with multiple peaks.

**Figure 2:**
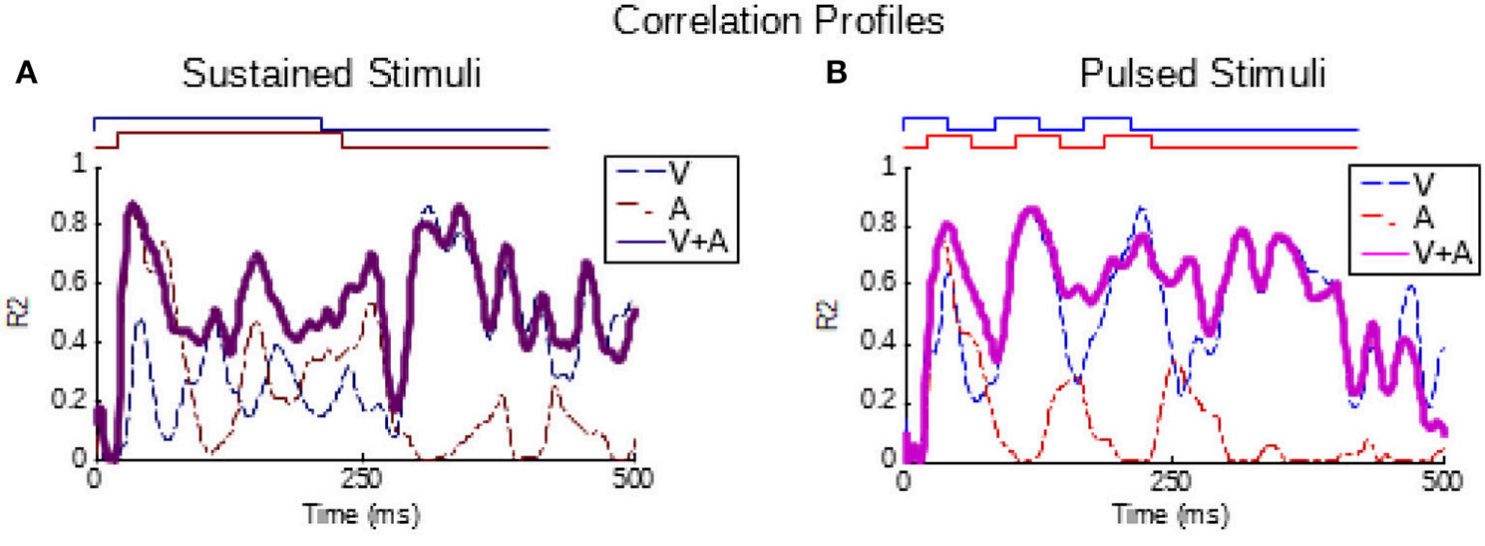
Partial correlations exist between the multisensory and component unisensory responses. Traces indicate the moment-by-moment correlation (*R*^2^) calculated across all samples between the multisensory responses and visual (blue, V), auditory (red, A), and summed unisensory instantaneous firing rate traces (purple, V+A). **(A,B)** Correlations for the sustained group are top-left, correlations for pulsed group are top-right. Note that the V+A correlation traces for both groups are significant throughout the response window, as reported for sustained stimuli by Miller et al. (2017).

As can be seen from Figure 1, the temporal structure of the modality-specific stimuli had a significant effect on the dynamics of the evoked unisensory responses. This is most clearly visible in the instantaneous firing rate traces on the bottom row. As illustrated in Figure 1, sustained visual and auditory stimuli often elicited a response with a single “on” component, sometimes an “off” component, and sometimes an elevated firing rate sustained throughout the response window. However, peak responses (measured in the instantaneous firing rate) were most frequently observed during the “on” or “off” phases. In contrast, pulsed stimuli over the same time window elicited multiple response components; i.e., multiple peaks. As illustrated in Figure 1, the timing of each peak was consistent with either an “on” or “off” component elicited by one of the 50 ms component pulses. The presence/absence of “on” or “off” response components was not controlled in this study and varied across neurons and sensory modalities. As predicted by Miller et al. (2017), the temporal dynamics of the multisensory responses were well-linked to these unisensory temporal dynamics. Multisensory responses were consistent with a principle of real time integration; that is, converging visual and auditory inputs appeared to be integrated as soon as they arrived at the target neuron without delay or wind-up (Miller et al., 2017). Consequently, at each millisecond in time after response onset, there was a good correlation across samples between the instantaneous firing rate of the multisensory response and the firing rates of the component unisensory responses that had been appropriately time-shifted by their relative stimulus onsets. This is illustrated for the sustained (i.e., VsAs) group in Figure 2A (mean *R*^2^ = 0.57, peak *R*^2^ = 0.87). The same high correlation at each moment in time was observed for the pulsed (i.e., VpAp) group (Figure 2B; mean *R*^2^ = 0.65, peak *R*^2^ = 0.86). Thus, the basic principles of the multisensory transform identified for sustained stimuli also apply to the integration of pulsed stimuli.

Enhanced multisensory responses were reliably elicited by these spatially concordant visual-auditory cross-modal stimuli, and were evident in these neurons regardless of whether the two modality-specific component stimuli were sustained, pulsed, or one was pulsed and the other sustained. In addition, the response magnitudes and distributions of ME and AI were similar and in keeping with prior evaluations of multisensory enhancement in this structure (response durations were longer, reflecting the longer stimulus durations used here, Yu et al., 2010; Xu et al., 2012; Miller et al., 2015). Despite these consistencies, there were significant differences between the level of enhancement observed for sustained and pulsed stimuli, as described below.

**Figure 3:**
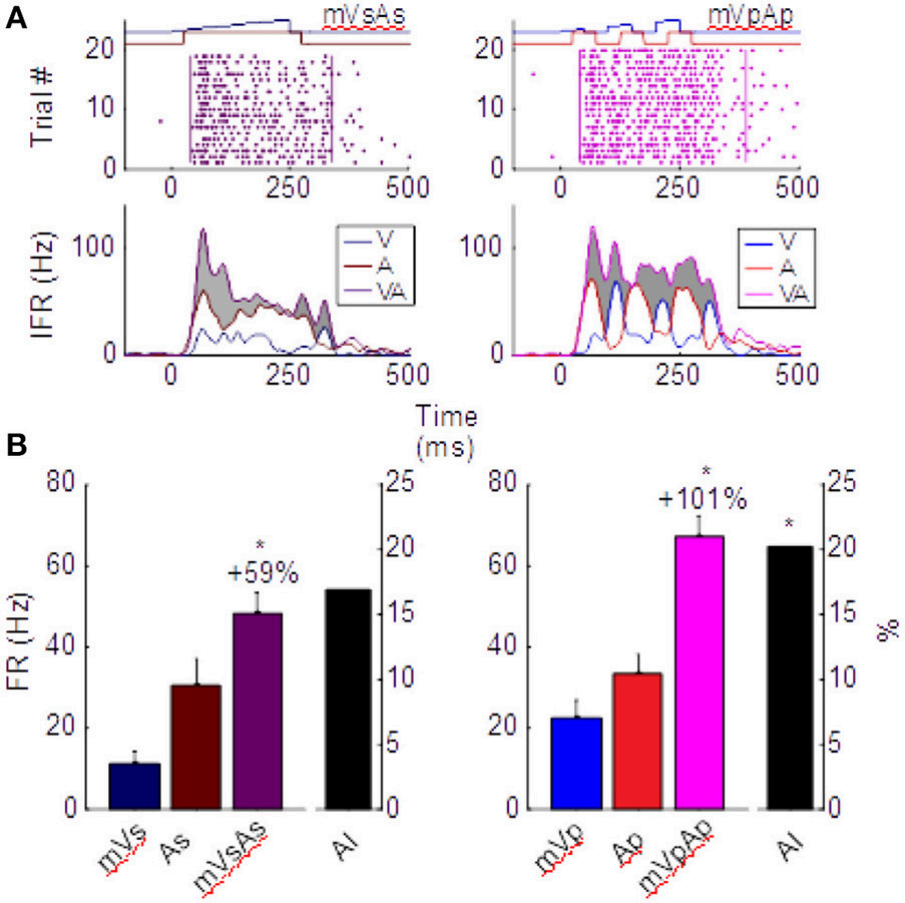
Different multisensory products are produced by pairs of sustained (left) and pulsed (right) stimuli in the same neuron. **(A)** Impulse rasters illustrate the multisensory responses of the neuron whose unisensory responses are shown in Figure 1. Those elicited by pairing the sustained moving visual and auditory cues (mVsAs) on the left can be compared to those elicited by pairing their pulsed counterparts (mVpAp) on the right. The instantaneous firing rate (IFR) traces for each of these multisensory responses (VA, purple) to the cross-modal pairing is plotted below together with the IFR traces of the responses evoked by the component visual (V, blue) and auditory (A, red) stimuli. Enhancement of the multisensory IFR over the largest unisensory IFR is shaded in gray. Note this enhancement is largely restricted to the initial portion of the response for the sustained cues, but continues throughout the response to the pulsed cues. This is a consequence of the apparent fusion of responses to the multiple pulses. **(B)** This leads to a larger proportionate multisensory enhancement (101 vs. 59%) and significantly more superadditivity (indicated by the AI metric) in the pulsed condition. Error bars indicate sem, ^*^p < 0.05.

One of the features of multisensory enhancement that is directly derived from its underlying transform is a phenomenon termed the “Initial Response Enhancement” (IRE). This is a large enhancement typically observed near the time at which inputs from the two cross-modal stimuli begin overlapping (Rowland and Stein, 2007, 2008; Rowland et al., 2007b; Miller et al., 2015, 2017). This early enhancement is typically superadditive and much more robust than enhancements observed later in the response, which generally become additive. This trend can be seen in the left column of Figure 3, which depicts the multisensory responses from the exemplar neuron whose modality-specific responses are illustrated in Figure 1. Evaluated over the entire response window, the multisensory response is significantly larger than the largest unisensory comparator response (ME = 59.2%, *p* < 0.01), but it is not superadditive (AI = 16.8%, *p* = 0.08). However, within the early response phase containing the IRE (*t* = [30,100] in Figure 3), the multisensory enhancement was slightly stronger (ME = 66%, *p* < 0.01), and more superadditive as well (AI = 31%, *p* < 0.01).

The important difference between the sustained and pulsed conditions is that by repeatedly engaging the multisensory transform, multiple IREs are generated. This is evident in the right column of Figure 3. The IRE for the first response component (in window *t* = [30, 100]) shows enhancement (ME = 64%, *p* < 0.01) and superadditivity (AI = 34%, *p* < 0.01) comparable to that observed in the IRE of the sustained condition on the left. However, the amplification associated with the second (in *t* = [100, 200], ME = 122%, AI = 19%) and third (in *t* = [200, 300], ME = 100%, AI = 18%) components are also each significantly enhanced (*p* < 0.01) and superadditive (*p* < 0.05). They support a period of robust response enhancement over a larger window. Consequently, multisensory enhancement measured over the entire response window is more robust and superadditive for pulsed stimuli (ME = 101%, *p* < 0.01; AI = 20.1%, *p* < 0.01) than sustained stimuli (Figure 3B).

**Figure 4:**
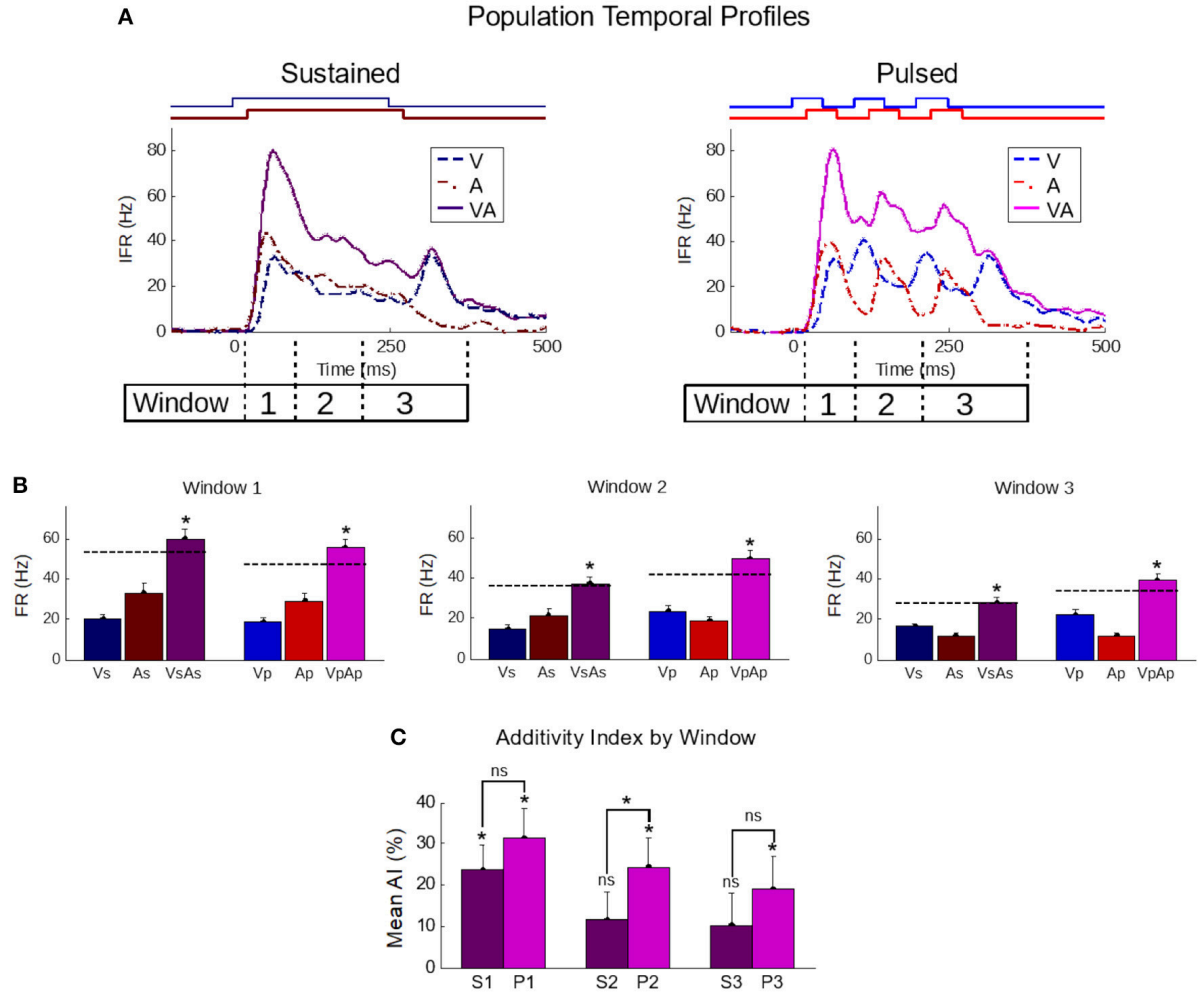
The temporal profile of population-averaged enhancement reveals the mechanisms underlying the different multisensory response products. **(A)** Population-averaged instantaneous firing rate profiles for responses to individual modality-specific component stimuli and their pairings in two conditions: when the stimuli were sustained (left) and when those same stimuli were pulsed (right). Paired sustained stimuli elicited a characteristic Initial Response Enhancement (IRE, Rowland et al., 2007b) that was superadditive, and diminished steadily over the course of the response. Paired pulsed stimuli (right) had multiple response peaks and thus, multiple periods of superadditive response enhancement. This reflected the overlap of the most dynamic periods of the repeated cross-modal inputs. **(B)** The effect is further quantified by calculating the mean firing rate within three windows of time (window 1 = 30–100 ms; window 2 = 100–200 ms; window 3 = 200–350 ms) corresponding to the early, middle, and late phases of the response. Dotted lines in the bar graphs indicate the summed unisensory firing rates. While the sustained multisensory responses (VsAs) quickly diminished, the pulsed multisensory responses (VpAp) remained far more robust throughout. Asterisks indicate significant enhancement (ME) **(C)** The average additivity index (AI) highlighted the transient nature of the superadditive initial response enhancement for response windows when the sustained stimuli were presented (S1-S3), and the superadditive enhancement observed in the same windows when the stimuli were pulsed (P1-P3). AI between the groups is significantly different in the second window. Conventions are the same as previous figures.

That pulsed stimuli elicited more robust and superadditive enhancement than sustained stimuli was consistently observed across the population (Figure 4). Population-averaged IFR traces across all samples in the sustained group confirmed the presence of the IRE (Figure 4, top-left). In contrast, averaged traces for samples in the pulsed group showed multiple cycles of enhancement (Figure 4, top-right). To highlight the impact of these differences quantitatively, responses were blocked into early (“Window 1”, *t* = [30,100]), middle (“Window 2”, *t* = [100,200]), and late (“Window 3”, *t* = [200,350]) phases of the response. These windows bracket the different components in the responses to the pulsed stimuli.

In the early phase (first window), multisensory responses are significantly enhanced and superadditive (*p* < 0.01) in both sustained (ME = 88.8 ± 11.5%; AI = 23.6 ± 6.1%) and pulsed (ME = 109 ± 13.5%; AI = 31.6 ± 6.8%) groups, with no significant differences in either metric between groups (*p*-value for ME = 0.13, *p*-value for AI = 0.2). Notably, the stimuli and temporal profiles of the responses in the different groups are very similar within this range.

However, in the subsequent response windows, enhancements elicited by sustained stimuli were often absent or showed a progressive decline. In the second window, the AI elicited by sustained stimuli was significantly decreased (*p* < 0.05) in additivity (11.6 ± 6.7%, *p* = 0.09). There was no significant change (*p* = 0.82) from the level of AI in the second to that observed in the third window (10.2 ± 7.8%, *p* = 0.2). This observed transition from early superadditivity in the IRE to additivity in later windows is highly consistent with prior observations (Rowland and Stein, 2007, 2008; Miller et al., 2015, 2017).

In contrast, response magnitudes to the pulsed stimuli were much more robust, maintaining levels more similar to IRE in later windows of time. There was not only significant mean enhancement in the magnitude of the multisensory response during these periods (i.e., window 2: 87.6 ± 12.5%, *p* < 0.01; window 3: 69.2 ± 12.1%, *p* < 0.01), but enhancements achieved superadditive levels as measured by AI (i.e., window 2: 24.2 ± 7.1%, *p* < 0.01; window 3: 19.0 ± 8.0%, *p* < 0.05). The marginal trend of decreasing AI over time was non-significant (*p* = 0.11); however, due to its presence and the existence of interneuronal variability, the AI difference between the sustained and pulsed conditions only reached statistical significance in the second window (*p* < 0.05) (Figure 4).

In short, in the population, as in the exemplar, the multiple cycles of enhancement elicited by pulsed cues combined to yield an overall level of enhancement (ME = 78.2%, AI = 19.3%) greater (*p* < 0.05) than that elicited by sustained cues (ME = 65.2%, AI = 10.5%).

The analysis of enhancement uses proportionate metrics (ME and AI) that provide some control over differences in the level of unisensory effectiveness between groups via normalization, with AI providing slightly better control (cf. Miller et al., 2015). This is typically an important control in comparisons between responses elicited by stimuli with different features. In the present dataset, the summed unisensory response magnitude were approximately equal in the pulsed and sustained conditions (*p* = 0.25). However, this similarity did not reflect a constancy in the visual and auditory response magnitudes; rather, it reflected different trends in the pulsed vs. sustained response magnitudes for the visual and auditory modalities. The average firing rates of the responses to pulsed visual stimuli (mean ± σ: 19.7 ± 13.7 Hz) were significantly (*p* < 0.01) greater than the responses to sustained stimuli (14.9 ± 9.2 Hz). Auditory firing rates, on the other hand, were significantly (*p* < 0.05) weaker for pulsed (16 ± 12.3 Hz) vs. sustained (17.7 ± 14.9 Hz) stimuli.

**Figure 5:**
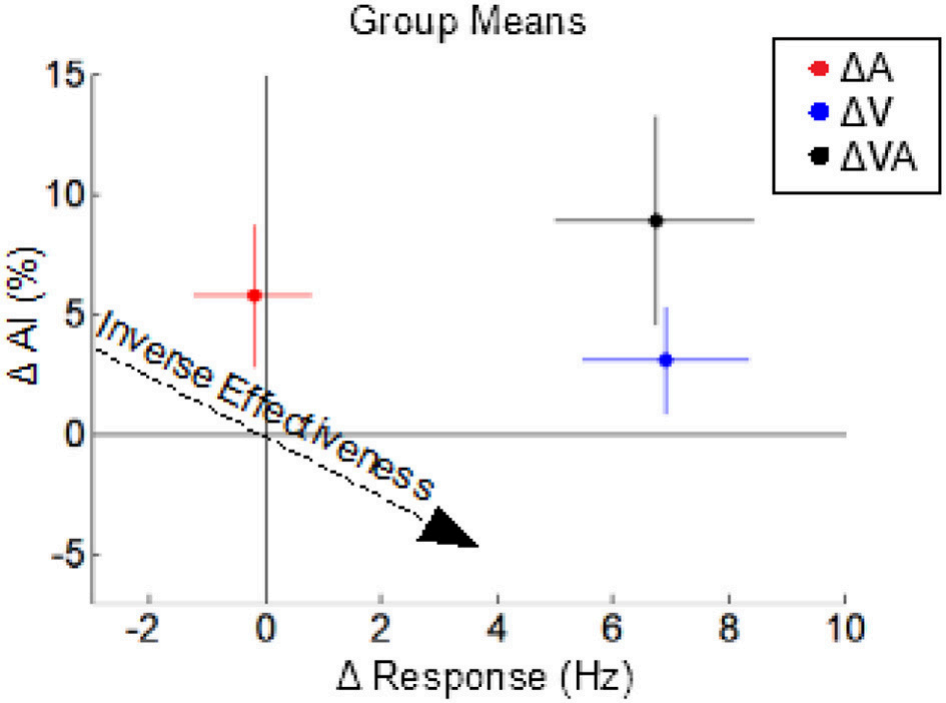
The effects of switching from sustained to pulsed cues on the multisensory product. Dots indicate the mean changes in multisensory response magnitude (x-axis) vs. changes in AI (y-axis) observed in three different comparisons: VpAp vs. VsAs (ΔVA, black), VpAp and VpAs vs. VsAp, and VsAs (ΔV, blue), and VsAp and VpAp vs. VsAs and VpAs (ΔA, red). Colored lines indicate sem for the groups. Switching from a sustained to a pulsed visual stimulus increases multisensory response magnitude but not (significantly) AI. Switching from a sustained to a pulsed auditory stimulus increases AI but not response magnitude. However, switching both from sustained to pulsed temporal structures yields the combined effects: an increase in both response magnitude and AI, which is opposite the trends predicted by the principle of inverse effectiveness (dashed arrow).

The multisensory effects (on response magnitude and AI) of switching each or both of the modalities from a sustained to a pulsed configuration were examined by appropriately grouping and comparing multisensory test conditions; for example, the effect of changing from a sustained to a pulsed visual configuration was obtained by grouping and comparing multisensory conditions in which the visual was sustained (VsAp and VsAs) to those in which it was pulsed (VpAp and VpAs). Changing the visual stimulus from a sustained to a pulsed configuration significantly (*p* < 0.01) increased the multisensory firing rate (Δ 6.9 ± 1.0 Hz) but had no significant effect (*p* = 0.17) on AI (Δ 3.1 ± 2.2%) (ΔV in Figure 5). In contrast, changing the auditory stimulus from a sustained to a pulsed configuration had no significant effect (*p* = 0.79) on the multisensory firing rate (Δ −0.2 ± 0.71 Hz) and a marginally non-significant (*p* = 0.054) effect on AI (Δ 5.8 ± 3.0%) (ΔA in Figure 5). The effect of changing both the visual and auditory stimuli from sustained to pulsed configurations was a significant increase (*p* < 0.01) in both the multisensory firing rate (Δ6.74 ± 1.0 Hz) and a significant increase (*p* < 0.05) in AI (Δ8.91 ± 4.34%) (ΔVA in Figure 5). Thus, co-pulsed stimuli elicit multisensory responses that are both *larger* and associated with *greater enhancement*/superadditivity than responses to co-sustained stimuli (Figure 5). It is important to note that both stimuli were required to switch from a sustained to a pulsed configuration to reveal the full effect. This combination of results is contrary to the well-documented *principle of inverse effectiveness*, whereby response magnitude and enhancement level are predicted to be *inversely* related to one another (Meredith and Stein, 1986). Note that in this case, the additivity index does not remain constant as response magnitude increases, but actually increases. We have previously noted that such violations may indicate cases of special interest (Stein et al., 2009). However, while such a violation is “surprising” in that it is rarely observed, here it was predictable given the applied principles identified for the multisensory transform from previous work (Miller et al., 2017).

## Discussion

Because the senses are in constant operation, so too is the multisensory process that integrates across them. Because this process can profoundly affect perception and behavior, a great deal of attention has been directed at understanding the circumstances under which this integrative process is initiated and the neural products that it yields. Recent efforts identified some of the crucial aspects of the underlying multisensory transform (Miller et al., 2017) in the SC, and some of the basic design constraints in this midbrain circuit. It also provided a basis for predicting how it will respond to stimulus dimensions not yet explored, such as those manipulated here.

An essential insight derived from understanding the multisensory transform is that it operates in continuous time on a moment-by moment basis. Looking at discrete time windows (generally response magnitudes across the entire duration of the response) does not capture the full complexity underlying the integration of multisensory inputs. The process of integration begins as soon as the unisensory inputs converge on their common SC target neuron, and it yields an ongoing, albeit changeable, multisensory output of varying duration. Thus, in a transparent way, manipulating the stimulus features to produce very different unisensory response profiles and alignments will produce correspondingly different multisensory outputs. Those that maximize the overlapping periods of unisensory excitation will then yield multiple periods of enhancement that can be observed during the temporal evolution of a multisensory response. As established by (Miller et al., 2017), this output (i.e., the integrated multisensory response) is directly related to the dynamics and relative alignment of the unisensory inputs that are integrated through the transform. Our present findings emphasize the applicability of this principle to more complex stimuli (temporal complexity of pulsed stimuli) given the high correlative nature between the summed unisensory responses and the observed multisensory product. This means that, in theory, it is possible to identify *a priori* sets of stimulus features that will produce more enhancement than others simply by knowing the dynamics of the unisensory responses they elicit. This would facilitate current strategies that use the principles of multisensory integration to rehabilitate sensory deficits (Bolognini et al., 2005; Leo et al., 2008; Passamonti et al., 2009; Dundon et al., 2015; Jiang et al., 2015; Hadid and Lepore, 2017).

The practical difference between the pulsed and sustained stimuli used in the current study, is that the latter contained only one “rising phase,” whereas the former contained three. As previously noted, proper alignment of the single rising phase of each of the two sustained stimuli produced corresponding unisensory inputs that were fused and transformed to yield a superadditive signal at the early stages of the multisensory response (the Initial Response Enhancement, IRE). Because the remainder of the response was of far lower magnitude, the IRE accounted for a large proportion of the overall enhancement (Rowland and Stein, 2007, 2008; Rowland et al., 2007b; Miller et al., 2015, 2017). Co-pulsed visual and auditory cues, by virtue of their multiple instances of aligned rising phases, produced multiple “IREs” and thus greater overall enhancement.

It is notable that the increase in multisensory enhancement observed for pulsed stimuli was obtained despite an *increase* in their unisensory inputs as well. In short, proportionate multisensory enhancement violates expectations based on the “principle of inverse effectiveness” (Meredith and Stein, 1986). This specifies that multisensory enhancement magnitudes are inversely related to the efficacy of the unisensory inputs: the stronger the inputs the smaller the proportionate ‘benefits' achieved by their integration. This principle has very broad support in the empirical literature (Stein et al., 2009), and is consistent with basic statistical reasoning. Its violation with pulsed stimuli reveals an important sensitivity of the system to the temporal patterning of its inputs. Since biological systems often elicit cross-modal stimuli with particular temporal patterns and rhythms, having a system particularly sensitive to them would make intuitive sense. Whether, in fact, the present observations reveal a system tuned to ecologically relevant stimulus patterns remains to be determined.

However, the findings confirm that the dynamics of the unisensory responses provide sufficient information to extrapolate the multisensory transform on a moment-to moment basis. Although they do not eliminate the possibility that the multisensory transform itself can be altered, it is not clear what mechanisms would have to be put in place to do so. Thus our findings further suggest that in order for other cross-modal stimulus features to be enhanced selectively to reliably violate inverse effectiveness, the changes would have to be made in their unisensory dynamics.

## Author contributions

EB, JV, BR, and BS: contributed to the conception and design of the work and wrote and/or edited the manuscript; EB and JV: performed experiments.

### Conflict of interest statement

The authors declare that the research was conducted in the absence of any commercial or financial relationships that could be construed as a potential conflict of interest.
